# Uncultured *Gammaproteobacteria* and *Desulfobacteraceae* Account for Major Acetate Assimilation in a Coastal Marine Sediment

**DOI:** 10.3389/fmicb.2018.03124

**Published:** 2018-12-18

**Authors:** Stefan Dyksma, Sabine Lenk, Joanna E. Sawicka, Marc Mußmann

**Affiliations:** ^1^Department of Molecular Ecology, Max Planck Institute for Marine Microbiology, Bremen, Germany; ^2^Faculty of Technology, Microbiology – Biotechnology, University of Applied Sciences, Emden, Germany; ^3^Department of Biogeochemistry, Max Planck Institute for Marine Microbiology, Bremen, Germany; ^4^Division of Microbial Ecology, Department of Microbiology and Ecosystem Science, University of Vienna, Vienna, Austria

**Keywords:** sulfate-reducing bacteria, *Desulfobacteraceae*, *Desulfobulbaceae*, *Gammaproteobacteria*, *Roseobacter*-clade bacteria, polyhydroxyalkanoates, flow cytometry

## Abstract

Acetate is a key intermediate in anaerobic mineralization of organic matter in marine sediments. Its turnover is central to carbon cycling, however, the relative contribution of different microbial populations to acetate assimilation in marine sediments is unknown. To quantify acetate assimilation by *in situ* abundant bacterial populations, we incubated coastal marine sediments with ^14^C-labeled acetate and flow-sorted cells that had been labeled and identified by fluorescence *in situ* hybridization. Subsequently, scintillography determined the amount of ^14^C-acetate assimilated by distinct populations. This approach fostered a high-throughput quantification of acetate assimilation by phylogenetically identified populations. Acetate uptake was highest in the oxic-suboxic surface layer for all sorted bacterial populations, including deltaproteobacterial sulfate-reducing bacteria (SRB), which accounted for up to 32% of total bacterial acetate assimilation. We show that the family *Desulfobulbaceae* also assimilates acetate in marine sediments, while the more abundant *Desulfobacteraceae* dominated acetate assimilation despite lower uptake rates. Unexpectedly, members of *Gammaproteobacteria* accounted for the highest relative acetate assimilation in all sediment layers with up to 31–62% of total bacterial acetate uptake. We also show that acetate is used to build up storage compounds such as polyalkanoates. Together, our findings demonstrate that not only the usual suspects SRB but a diverse bacterial community may substantially contribute to acetate assimilation in marine sediments. This study highlights the importance of quantitative approaches to reveal the roles of distinct microbial populations in acetate turnover.

## Introduction

Acetate is an important intermediate in the turnover of organic matter in most aquatic sediments (Reeburgh, 1983). It is an end product of anaerobic fermentation, but also many aerobic microbes release acetate during imbalanced growth conditions. The removal and mineralization of this key metabolite is vital to carbon cycling and drives central anaerobic respiration processes in aquaticbenthic ecosystems. In marine sediments pore water acetate typically occurs in the micromolar range and is quickly consumed (Sørensen et al., 1981; Christensen, 1984; Parkes et al., 1989; Kristensen, 1994; Finke et al., 2007; Valdemarsen and Kristensen, 2010). While acetate can also be assimilated for biosynthesis, it is one of the most important substrate powering anaerobic sulfate respiration, a process accounting for up to 50% of total carbon mineralization in shelf sediments (Jørgensen, 1982; Oremland and Polcin, 1982; Werner et al., 2006). Acetate also drives metal-dependent respiration in anoxic, iron- and manganese-rich marine sediments (Christensen and Blackburn, 1982; Reeburgh, 1983; Novelli et al., 1988; Vandieken and Thamdrup, 2013).

To identify acetate-consuming microorganisms, stable isotope probing (SIP) has been frequently used to track assimilation of isotopically labeled model substrates into cellular biomarkers such as nucleic acids (DNA/RNA-SIP) or phospholipid fatty acids (PLFA-SIP) (Boschker et al., 1998; MacGregor et al., 2006; Webster et al., 2006, 2010; Miyatake et al., 2009; Vandieken et al., 2012; Vandieken and Thamdrup, 2013; Na et al., 2015). Due to the central role of acetate for sulfate reduction most studies so far focused on the identification of acetate-consuming sulfate reducing bacteria (SRB). The deltaproteobacterial *Desulfobacteraceae* have been repeatedly detected in SIP approaches (Boschker et al., 1998; Webster et al., 2006, 2010; Na et al., 2015) but quantitative data such as cellular assimilation rates or relative contribution to total acetate assimilation are lacking. Other deltaproteobacterial SRB such as the *Desulfobulbaceae* also occur ubiquitously and are highly abundant in marine sediment (Rabus et al., 2015), but *in situ* acetate consumption in this family has not been demonstrated. Besides SRB, other phylogenetic groups have been implicated in acetate assimilation such as Firmicutes, Crenarchaeota, and *Gammaproteobacteria* (Boschker et al., 1998; Webster et al., 2006, 2010; Miyatake et al., 2009; Seyler et al., 2014; Na et al., 2015). Diverse *Gammaproteobacteria* and the *Arcobacter* (*Campylobacter*) respire acetate with oxygen, nitrate or metal oxides as electron acceptors (Vandieken et al., 2012; Vandieken and Thamdrup, 2013).

In SIP the long incubation periods ranging from days to a few weeks can introduce well described biases such as cross-feeding and population shifts (Dumont and Murrell, 2005). Moreover, microbes that assimilate isotopic labels into compounds other than nucleic acids or PFLAs generally escape detection by SIP. Most importantly, the uptake of an isotopically labeled model compound by a discrete population cannot or only indirectly be quantified in such approaches. Thus, we still know little about the relative contribution of individual microbial populations to acetate assimilation in marine sediments.

Goal of our study was to quantify the assimilation of acetate by distinct bacterial populations during short-term incubations in tidal sediments. For this purpose we applied a novel methodological approach to quantify the assimilation of radiolabeled (^14^C) acetate by combining fluorescence *in situ* hybridization (FISH), flow sorting and scintillography (Dyksma et al., 2016). We focused on selected, *in situ* abundant and ubiquitous bacterial populations including *Gammaproteobacteri*a, *Roseobacter*-clade bacteria (RCB) and the sulfate-reducing *Desulfobulbaceae* and *Desulfobacteraceae*. In addition, we flow-sorted cells containing fluorescently stained polyhydroxyalkanoates (PHAs) which allowed us to quantify the assimilation of ^14^C-acetate into storage compounds.

## Materials and Methods

### Study Site and Sampling

In June and October 2009 at low tide sediment was sampled by 3.7 cm diameter polyacryl cores at the tidal flat Janssand (53.7366N, 7.6989E) located in the German Wadden Sea. Biogeochemical data and sediment characteristics of this site are published elsewhere (Billerbeck et al., 2006; Beck et al., 2009; Jansen et al., 2009; Kowalski et al., 2012). Sediment cores of 20 cm length were taken, stored in the dark at *in situ* temperatures (15–20°C) and processed within 24–48 h after sampling.

### Pore Water Concentrations of Acetate

Concentrations of acetate were measured in two sediment cores sampled in June 2009. High-performance liquid chromatography (HPLC), collection and analysis of pore water were performed as described by Sawicka et al. (2010). In brief, sediment samples from respective depths were centrifuged in SphinexR 135 filters at 4000 rpm at 4°C for 15 min. Collected pore water was filtered into 1-ml brown borosilicate glass vials that were pre-combusted at 480°C for 4 h to minimize possible contamination. The acids were derivatized with *p*-nitrophenyl hydrazine, separated by HPLC using a LiChrospher 80/100 (Knauer, Berlin, Germany) column at 25°C, and the concentrations were determined from the absorption on a UV/VIS detector (Linear) at 400 nm.

### Whole Sediment Core and Slurry Incubations With ^14^C-Acetate

Intact sediment cores from the same sampling campaign in June 2009 were percolated with 50 ml of sterile filtered, oxic pore water containing 100 μM [1,2-^14^C]-acetate (specific activity 100 mCi/mmol, Hartmann Analytic, Braunschweig, Germany) to displace pore water as described by Lenk et al. (2011) and incubated for 8 h in the dark at *in situ* temperature (20°C). After incubation, sediment cores were washed twice with sterile filtered seawater (50 ml) to remove free ^14^C-acetate and were sliced into 1 cm sections. From each layer 0.5 ml were fixed for FISH, flow-sorting and microautoradiography (MAR) as described previously (Lenk et al., 2011).

In October 2009 we prepared sediment slurries from 0 to 1 cm sediment depth for oxic and anoxic (N_2_-gassed) incubations. We added 100 μM [1,2-^14^C]-acetate to 1 ml of sediment and 1 ml of sterile-filtered seawater and incubated at 200 rpm. The slurries were incubated in 5 ml vials for 8 h at 20°C. All sediment samples were fixed by adding 1.8% formaldehyde overnight at 4°C and processed for CARD-FISH analysis according to Ishii et al. (2004). Cells were subsequently either labeled by CARD-FISH or Nile Red staining for flow-sorting. Anoxic sediment slurries were used for Nile red staining only. Formaldehyde-inactivated sediment served as control for unspecific adsorption of acetate to cells or particles and showed only minimal ^14^C incorporation.

### Microautoradiography (MAR)

The relative abundance of ^14^C-acetate-assimilating cells in sediment cores from June 2009 was determined by MAR. MAR was performed according to Alonso and Pernthaler (2005) and Lenk et al. (2011) with an exposure time of 2 days. Relative abundance of MAR-positive cells was manually determined under an Axioplan epifluorescence microscope (Zeiss, Jena, Germany).

### Measurements of Acetate Uptake by the Bulk Microbial Community

Bulk uptake of acetate by the microbial community in core incubations and slurry experiments was measured in formaldehyde-fixed cells separated from sediment particles. For this purpose, cells were detached from sediment grains by ultrasonic treatment as described previously (Lenk et al., 2011). After sonication 10 μl of the supernatant containing the detached cells was mixed with 5 ml Ultima Gold XR (PerkinElmer, Boston, MA, United States) scintillation cocktail and the radioactivity was measured in a liquid scintillation counter (Tri-Carb 2900, PerkinElmer, United States). Scintillation counts of formaldehyde-inactivated dead controls were minimal and were subtracted from values measured in live incubations.

### CARD-FISH and Sample Preparation for Flow Cytometry

For catalyzed reporter deposition-fluorescence *in situ* hybridization (CARD-FISH) sediment was processed as described in Lenk et al. (2011). Directly after ultrasonication supernatants were filtered onto 25 mm polycarbonate membrane filters with a 0.2 μm pore size (GTTP, Millipore, Eschborn, Germany). Permeabilization and CARD-FISH was performed as described by Pernthaler et al. (2002) but without embedding in agarose and with the following modifications. Endogenous peroxidases were inactivated in 3% H_2_O_2_ for 10 min at room temperature. The hybridization temperature was 46°C and washing was performed at 48°C according to the protocol of Ishii et al. (2004). An overview of oligonucleotide probes used in this study is shown in Supplementary Table S1. Tyramides labeled with Alexa488 fluorescent dye (Molecular Probes, United States) were used for CARD signal amplification. Cells were removed from filter membranes using a cell scraper or were vortexed in 5 ml of 150 mM NaCl containing 0.05% Tween 80 according to Sekar et al. (2004). Prior to flow cytometry, large suspended particles were removed by filtration through 8 μm pore-size filter (Sartorius, Göttingen, Germany) to avoid clogging of the flow cytometer.

### Fluorescence Activated Flow Sorting (FACS) and Scintillography of Sorted Cells

Flow sorting of FISH-identified cells and scintillation counting of sorted cell fractions were performed as described previously (Dyksma et al., 2016). In brief, cells were sorted using a FACSCalibur flow cytometer (Becton Dickinson, Oxford, United Kingdom). Hybridized cells were identified on scatter dot plots of green fluorescence versus 90° light scatter and sediment background was determined by flow cytometric analysis of hybridizations with a nonsense probe (NON338) prior to flow sorting. Collected cell batches on polycarbonate filters were transferred into 5 ml scintillation vials and mixed with 5 ml Ultima Gold XR (PerkinElmer, Boston, MA, United States) scintillation cocktail. Radioactivity of sorted cell batches was measured in a liquid scintillation counter (Tri-Carb 2900, PerkinElmer, United States). Radioactive background by unspecifically adsorbed ^14^C-acetate was determined by spiking experiments with fluorescent beads (Polyscience Fluoresbrite^®^ Yellow Green Microspheres, 1 μm) and was only minor (Supplementary Figure S1).

For measuring assimilation of ^14^C-acetate into PHAs we sampled oxic and anoxic sediment slurries prepared from 0 to 1 cm depth sampled in October 2009 (see above). For Nile red staining of PHAs, cells were detached from the sediment as described above and stained with 10 μg/ml (final concentration) Nile red from a stock solution of 1 mg/ml dissolved in dimethylsulfoxide (DMSO). Cells were stained for 30 min at 37°C and a fraction of the stained sample was manually checked under an epifluorescence microscope (Zeiss, Jena, Germany). Prior to flow cytometry large suspended particles were removed by filtration through 5 μm pore-size filters (Sartorius, Göttingen, Germany). Target cells for flow cytometry were identified on scatter dot plots of orange fluorescence (detected wavelength 585 ± 21 nm) versus 90° light scatter. Regions with four different fluorescence intensities were selected for sorting (Supplementary Figure S2). Sorted cell batches were collected on polycarbonate filters and radioactivity was measured as described above.

### 16S rRNA Gene Phylogeny of Sva0081-MBG and Probe Design

We previously constructed a consensus-tree including *Desulfobacteraceae* and sequences of the Sva0081-MBG retrieved from our sampling site (for details see Dyksma et al., 2017). The probe design-tool implemented in the ARB program package (Ludwig et al., 2004) was used to design a specific probe for those sequences >1,200 bp of the Sva0081-MBG that grouped with the only partial sequences recovered from site Janssand (Supplementary Figure S3). As positive control for probe DSS1431 we used cross-sections of the marine gutless oligochaete *Olavius algarvensis* harboring the delta1 symbiont (kindly provided by N. Dubilier and M. Schimak, MPI Bremen). Helper and competitor probes were applied at 30% formamide in the hybridization buffer (Supplementary Table S1), yielding an optimal signal-to-noise-ratio in the tested sediment from site Janssand. Double-hybridizations with probe DSS658 targeting most members of the *Desulfosarcina*/*Desulfococcus*-group within the *Desulfobacteraceae* was performed to ensure specificity. Therefore, consecutive CARD-FISH using Alexa488- and Alexa546-labeled tyramides were performed with an intermediate inactivation of HRP-enzyme after the probe applied first. All FISH signals for probe DSS1431 overlapped with signals of probe DSS658. Unspecific signals were below 0.1% of total cell counts and were neglectable.

### Calculation of Average Cell-Specific Carbon Assimilation Rates

The average cell-specific carbon assimilation rates (fg C cell^-1^ d^-1^) were calculated from bulk measurements for a sorted population according to the equation *R* = *A* ×*M*/ *a* ×*n* ×*t* ×*L*. *A* represents the activity of the sorted cell batch in Becquerel (Bq), *M* represents the molar mass of carbon (g/mol), *a* equals the specific activity of the tracer (Bq/mol), *n* represents the number of sorted cells, *t* represents the incubation time in hours and *L* equals the ratio of total acetate/^14^C-acetate.

## Results

### Bulk Acetate Assimilation and Relative Abundance of Acetate-Assimilating Cells

To quantify bulk acetate assimilation and assimilation by distinct *in situ* abundant bacterial populations in a tidal sediment, we percolated two sediment cores with pore water containing 100 μM of [1,2-^14^C]-labeled acetate. This amount roughly equalled the 12-fold maximum acetate concentrations measured in the top 10 cm of the sediments (Supplementary Figure S4) and were added in order to prevent depletion of acetate, e.g., through potential adsorption to particles (Christensen and Blackburn, 1982). Seasonal variations and concentration profiles of electron acceptors such as oxygen, nitrate and metal oxides at our study site have been reported in detail (Billerbeck et al., 2006; Jansen et al., 2009; Kowalski et al., 2012). Our whole core incubations mimicked the stagnant conditions during low tide promoting a quick oxygen depletion already in few mm depth (de Beer et al., 2005; Jansen et al., 2009) and provided suitable conditions for both aerobic and anaerobic acetate uptake. According to previous microelectrode measurements (de Beer et al., 2005; Jansen et al., 2009), the surface layer from 0 to 1 cm depth in our sediments comprised both oxic and suboxic layers.

**FIGURE 1:**
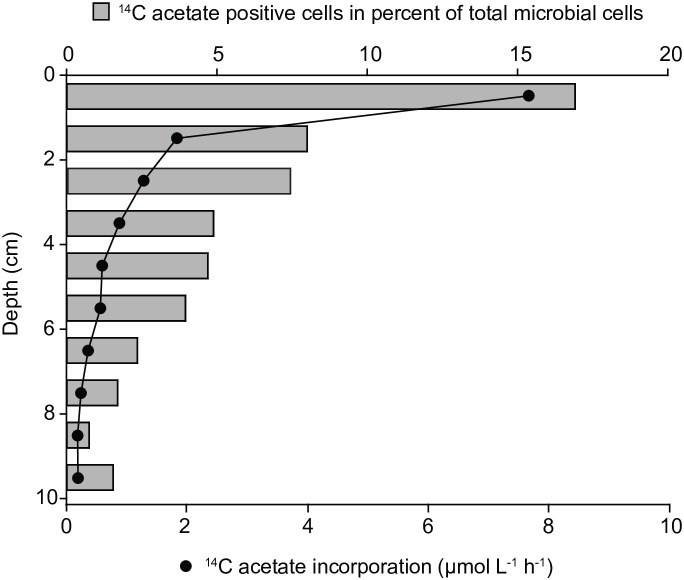
Bulk ^14^C-carbon assimilation and relative abundance of ^14^C-acetate-assimilating cells determined by microautoradiography after 8 h-incubations of whole sediment cores (June 2009).

In both sediment cores bulk acetate assimilation was highest the surface layer (0–1 cm) reaching up to 7.6 μmol l^-1^ h^-1^ and steeply decreased to 0.2 μmol l^-1^ h^-1^ in 9–10 cm sediment depth (Figure 1). The relative abundance of ^14^C-acetate-assimilating cells was determined by microautoradiography (MAR) of DAPI-stained cells. Consistent with bulk acetate assimilation, the relative abundance of acetate-assimilating cells peaked in 0–1 cm depth, equalling 17% of total cell counts (TCC) and decreased to 2% of TCC in 9–10 cm depth (Figure 1). Acetate-assimilating cells in the surface layer were further phylogenetically identified using MAR-FISH targeting taxa abundant in marine coastal sediments (Dyksma et al., 2016). *Gammaproteobacteria* accounted for about 60% of all MAR-positive cells, whereas Deltaproteobacteria and alphaproteobacterial Rhodobacteraceae/*Roseobacter*-clade bacteria (RCB) made up only for 6 and 7%, respectively (Supplementary Table S2). Approximately 19% of *Gammaproteobacteria*, 40% of RCB and 8% of Deltaproteobacteria displayed a MAR-signal (Supplementary Table S2).

### Quantification of Acetate Assimilation in FISH-Identified Bacterial Populations

Since MAR-FISH does not allow to exactly quantify the amount of assimilated, isotopically labeled acetate we applied a novel approach to determine the average amount of ^14^C-acetate assimilated by cells of a given phylogenetic group. For this purpose we purified 50,000 potentially ^14^C labeled cells from the incubated sediment using fluorescence activated cells sorting (FACS) after either CARD-FISH or Nile Red staining. Subsequently, these flow-sorted batches of 50,000 cells each were used for quantitative scintillography.

**FIGURE 2:**
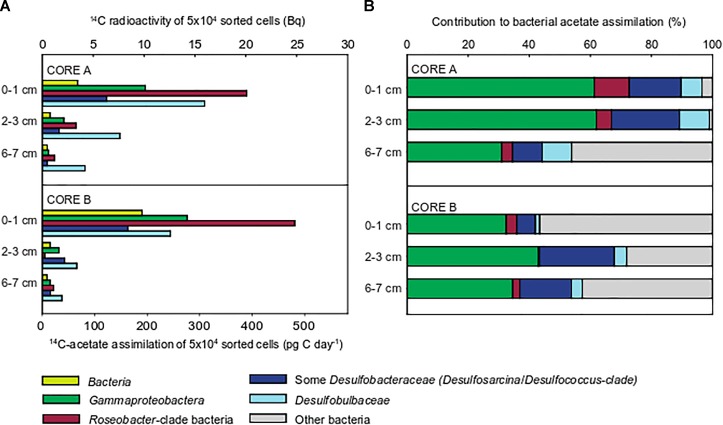
Acetate assimilation rates and ^14^C-carbon activity in 50,000 cells flow-sorted after CARD-FISH from incubations of whole sediment cores (June 2009). The following populations were flow-sorted according to their CARD-FISH signal: Bacteria (probe EUBI-III), *Gammaproteobacteria* (probe GAM42a), *Roseobacter*-clade bacteria (RCB, probe ROS537), *Desulfobulbaceae* (probe DSB706), and members of the *Desulfobacteraceae* (probe DSS658). Batches of 50,000 cells were sorted for quantification **(A)**. Integration of relative cell abundances of flow-sorted populations and their ^14^C-acetate assimilation rates shows the relative contribution to total bacterial ^14^C-acetate assimilation **(B)**.

For quantification of ^14^C acetate uptake, we targeted Bacteria, *Gammaproteobacteria*, RCB and members of the deltaproteobacterial, sulfate-reducing families *Desulfobacteraceae* and *Desulfobulbaceae* by CARD-FISH (for details on applied probes see Supplementary Table S1). Consistent with bulk measurements (Figure 1) all targeted populations displayed highest acetate uptake in the upper 0–1 cm, oxic-suboxic sediment layer and lowest uptake in 6–7 cm sulfidic layer (Figure 2). The ^14^C radioactivity measured in 50,000 sorted cells of the domain Bacteria ranged between 9.8 Bq in the uppermost cm to 0.5 Bq at 6–7 cm sediment depth.

*Roseobacter*-clade bacteria displayed highest assimilation rates of all populations amounting to 485 pg C d^-1^ in 50,000 cells (Figure 2). The measured assimilation rates for *Gammaproteobacteria* were approximately half as a much (275 pg C d^-1^). Likewise, the anaerobic sulfate-reducing *Desulfobacteraceae* and *Desulfobulbaceae* also displayed highest acetate assimilation in the oxic-suboxic surface layer, which even exceeded those in fully anoxic, sulfidic sediment layers. The *Desulfobulbaceae* assimilated acetate up to 355 pg C d^-1^, while *Desulfobacteraceae* showed lower rates of up to 165 pg C d^-1^.

Relative cell abundances of the individual target populations were highly similar throughout the upper 7 cm in both cores (Supplementary Figure S5), reflecting the extensive reshuffling of sediments, e.g., during tidal cycles. The community was dominated by *Gammaproteobacteria* (20–25% of TCC), followed by *Desulfobacteraceae* (7–10%), *Desulfobulbaceae* and RCB (1–2% each).

Integrating relative cell abundances with the uptake per sorted cell population allowed us to assess the relative contribution of individual populations to total bacterial acetate assimilation. *Gammaproteobacteria* contributed most to total bacterial ^14^C acetate uptake in the three sediment layers (31–62%, Figure 2). Although *Gammaproteobacteria* displayed low acetate assimilation rates per sorted population in the anoxic, sulfidic layer, they still accounted for most of ^14^C acetate uptake among the studied populations because of their higher cell abundances. RCB showed highest ^14^C-acetate assimilation per population, but contributed only 1–11% to bacterial acetate assimilation due to their low relative cell abundances (1–2% of TCC). The relative contribution of *Desulfobulbaceae* to total acetate assimilation slightly increased with sediment depth, while this was unclear for *Desulfobacteraceae*. Together, SRB contributed up to 32% of total bacterial acetate assimilation (Figure 2).

### Sediment Slurry Incubations

**FIGURE 3:**
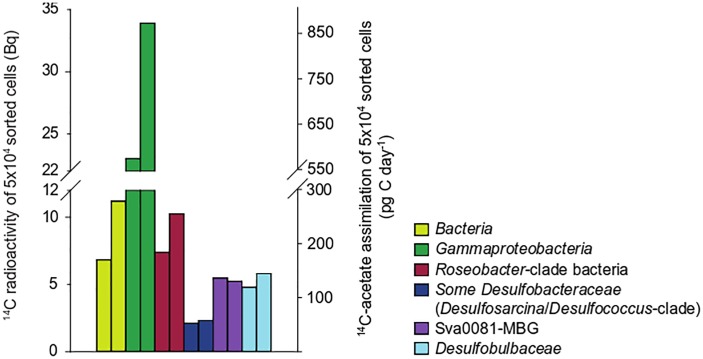
Acetate assimilation rates and ^14^C-carbon activity in 50,000 cells flow-sorted after CARD-FISH from duplicate sediment slurries incubated under oxic conditions (October 2009).

To confirm the observed differences in the cellular acetate uptake rates between the target populations, we returned to the study site in October 2009 and prepared oxic slurries with sediments recovered from 0 to 1 cm depth. Slurries were incubated at 100 μM ^14^C-acetate for 6 h. In contrast to core incubations from June 2009, *Gammaproteobacteria* displayed a threefold higher cellular acetate uptake than RCB (Figure 3). SRB assimilated less acetate than in surface layers in June 2009, but in agreement with core incubations the *Desulfobulbaceae* assimilated in average more acetate per sorted population than the *Desulfobacteraceae* (Figures 2, 3 and Supplementary Table S3).

Because of the pivotal role of *Desulfobacteraceae* in acetoclastic sulfate respiration in marine sediments, we took a closer look at an abundant subgroup within the *Desulfobacteraceae*, the uncultured Sva0081-marine benthic group (MBG) (Supplementary Figure S3). This group accounted for a major fraction of 16S rRNA amplicons affiliating with SRB at site Janssand (Dyksma et al., 2017) but acetate consumption in the Sva0081-MBG has not been demonstrated yet. Using partial and full-length 16S rRNA gene sequences we designed a specific FISH probe (Supplementary Table S1) to quantify acetate assimilation in the Sva0081-MBG in a nested approach. In oxic sediment slurries they displayed an acetate assimilation rate that was twice as high as the average rate of all targeted *Desulfobacteraceae* and was similar to rates measured in *Desulfobulbaceae* (Figure 3).

### Polyhydroxyalkanoate Synthesis From Acetate (PHA)

**FIGURE 4:**
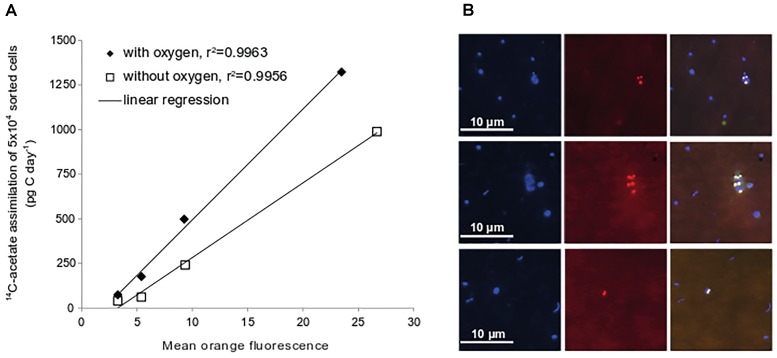
Acetate assimilation rates of flow-sorted cells after Nile Red-staining. Four different mean fluorescence intensity classes were defined for flow-sorting (see Supplementary Figure S2). Values are depicted as average of duplicate sediment slurries incubated under oxic or anoxic conditions (October 2009) **(A)**. Epifluorescence images of samples from sediment slurry incubations stained with DAPI and Nile Red. DAPI-stained cells, left panel; Nile Red-stain, middle panel; overlay of images right panel **(B)**.

In many organic-rich ecosystems with fluctuating oxygen concentrations microorganisms do built up storage compounds such as polyhydroxyalkanoates (PHA) from acetate (Anderson and Dawes, 1990). To test whether microorganisms in Janssand sediments also use acetate to synthesize PHA, we stained sediments from slurry incubations with the lipophilic, fluorescent dye Nile Red. We observed strongly fluorescent intracellular inclusions that were not found in controls (Figure 4). As the intracellular PHA concentration and fluorescence intensity of Nile Red correlate linearly (Greenspan et al., 1985; Vidal-Mas et al., 2001), we quantified fluorescence and thereby PHA in flow-sorted, Nile Red-stained cells. We defined four fluorescence intensity classes (Supplementary Figure S2) and measured ^14^C radioactivity in batches of 50,000 cells of each class. Fluorescence intensity of Nile Red stained cells correlated well with ^14^C radioactivity, indicating that these formed PHAs from acetate (Figure 4). Populations with the highest intracellular PHA concentration showed carbon assimilation rates up to 1,320 pg C d^-1^ in 50,000 cells (Figure 4), which exceeded all uptake rates measured for the phylogenetically identified populations.

## Discussion

In this study, we combined radioactive labeling of bacterial cells with CARD-FISH and flow-sorting to quantify the average cell-specific assimilation rates and the contribution to overall acetate assimilation of phylogenetically identified populations. The scintillography of 50,000 cells per measurement boosted sensitivity and accuracy as cells labeled below detection limits of techniques such as nanoSIMS or MAR-FISH also contribute to a single data point. On the downside our approach does not provide the phylogenetic resolution of qualitative studies tracking the incorporation of labeled isotopes into RNA/DNA and is limited by the available FISH probe sets. However, the increased sensitivity shortens incubations periods to 8 h or less and reduces artifacts inherent to SIP studies with commonly much longer incubation periods. Together, this approach allows a high sample throughput and a precision at the level of populations that complements existing isotope-labeling methods (Boschker et al., 1998; Nielsen and Nielsen, 2002; Vandieken et al., 2012; Vandieken and Thamdrup, 2013; Na et al., 2015).

Here, we demonstrate that the microbial community displayed a generally higher acetate-assimilating activity at the upper oxic-suboxic sediment layer than in deeper anoxic, sulfidic layers. This agreed well with highest bulk acetate uptake and relative abundances of MAR-FISH positive cells in the surface layer. Although cell-specific uptake rates are often highly divergent even within clonal populations we calculated average cell-specific C uptake rates from our data in order to compare our data with previous, semi-quantitative studies. For this purpose we divided the uptake rates of individual populations by the number of sorted cells (50,000) (Supplementary Table S3). The fraction of MAR-positive signals in a given population was not considered. According to this calculation the highest average cell-specific acetate assimilation rates amounted to 9.7 fg C cell^-1^ d^-1^ in the oxic-suboxic layer (Supplementary Table S3) and are in the lower range of rates measured for freshwater bacterioplankton (5–57 fg C cell^-1^ d^-1^; Buck et al., 2009). The vertical decrease in acetate uptake activity is consistent with earlier observations by Christensen and Blackburn (1980) and reflect that the turnover of organic matter in marine sediments generally decreases with sediment depth (Middelburg, 1989).

### Sulfate-Reducing *Desulfobacteraceae* and *Desulfobulbaceae* Assimilated Acetate

Our observation of high uptake rates by SRB in the oxic-suboxic surface layer and in the oxic sediment slurries was counterintuitive, since SRB are generally considered to thrive optimally under fully anoxic conditions (Rabus et al., 2015). However, there is accumulating evidence that sulfate reduction rates can be high in oxic-suboxic, sulfide-free habitats, when oxygen diffusion and consumption are balanced (de Beer et al., 2005; Bosselmann, 2007; Al-Raei et al., 2009; Wilbanks et al., 2014). Moreover, the increased availability of nutrients in the surface layer may stimulate sulfate reduction and mask the inhibitory effect of oxygen. Some SRB even grow slightly in presence of oxygen (reviewed in Rabus et al., 2015). Furthermore, anoxic microniches may provide a spatial niche for SRB in oxic sediments (Jørgensen, 1977). Future experiments require higher spatial and temporal resolution and defined incubation conditions to test for acetate uptake by SRB under different oxygen concentrations.

Here, we also show that members of the deltaproteobacterial order *Desulfobacterales* assimilate acetate in substantial amounts under close to *in situ* conditions and account for up to 32% of total bacterial acetate assimilation. While acetate assimilation by *Desulfobacteraceae* has previously been detected in qualitative SIP assays (Na et al., 2015), *Desulfobulbaceae* in marine sediments have so far not been reported to assimilate acetate, although cultured representatives use acetate as carbon source (Rabus et al., 2015). Our approach, however, indicated that the *Desulfobulbaceae* displayed even higher assimilation rates than the *Desulfobacteraceae* both in whole core and in slurry experiments. Among the *Desulfobacteraceae* the Sva0081-MBG assimilated acetate in higher rates than the average *Desulfobacteraceae* population (Figure 3), while in arctic sediment, this group was reported to assimilate only small amounts of acetate into biomass (Müller et al., 2018). Interestingly, the Sva0081-MBG is consistently found in high cell abundances in Wadden Sea sediments (up to 8% of total cell counts, Ovanesov, 2012) and has been frequently detected in various types of marine sediments worldwide (Wang et al., 2013; Liu et al., 2014; Zheng et al., 2014; Dyksma et al., 2017; Probandt et al., 2017; Müller et al., 2018). It also appears to be important for anaerobic hydrogen oxidation at our study site (Dyksma et al., 2017). Taken together, we provide first quantitative data supporting the vital role of the diverse *Desulfobacteraceae* including the Sva0081-MBG in carbon cycling in organic-rich marine sediments.

### *Gammaproteobacteria* Assimilate Major Amounts of Acetate

It was unexpected that *Gammaproteobacteria* consistently assimilated more acetate than the studied SRB, since concentrations of alternative electron acceptors such as metal oxides and nitrate in Janssand pore waters are usually in the lower micromolar range (Billerbeck et al., 2006; Gao et al., 2010; Kowalski et al., 2012) and thus far lower than those measured in coastal sediments that are dominated by, e.g., manganese-dependent carbon mineralization (Vandieken and Thamdrup, 2013). Although oxygen is usually quickly consumed by biotic and abiotic processes within minutes (de Beer et al., 2005), we cannot fully exclude that residual oxygen from percolated pore water stimulated an oxygen-dependent acetate uptake by *Gammaproteobacteria* in the deeper, sulfidic layers. Additionally, acetate can be assimilated into, e.g., PHAs even without respiratory activity using instead the reducing power from stored glycogen as observed in periodically anoxic activated sludge (Liu et al., 1994).

We could not identify these acetate-assimilating *Gammaproteobacteria* in more detail, but our previous studies show that facultatively anaerobic, sulfur-oxidizing members account for a major fraction of *Gammaproteobacteria* in Janssand and many other coastal sediments worldwide (Dyksma et al., 2016; Mußmann et al., 2017). Interestingly, other sulfur-oxidizing *Gammaproteobacteria* closely related to those identified at our study site have been shown to utilize acetate for the synthesis of substantial amounts of PHA (Kleiner et al., 2012; Flood et al., 2015). The hypothesized PHA synthesis from acetate by these *Gammaproteobacteria* is further supported by metatranscriptomic data from our study site. Transcripts encoding PHA synthases ranked among the top 1% expressed genes in distinct metagenomic bins of facultatively denitrifying *Gammaproteobacteria* including the Woeseiaceae/JTB255-MBG (Mußmann et al., 2017; Pjevac and Mußmann, unpublished data). Furthermore, our data are consistent with qualitative SIP approaches suggesting that in some marine sediments diverse *Gammaproteobacteria* incorporate acetate probably with oxygen, nitrate, or metals as electron acceptors (Vandieken et al., 2012; Vandieken and Thamdrup, 2013). Together, our findings support the notion that processes other than sulfate respiration and methanogenesis are important for carbon mineralization in marine sediments (Canfield et al., 1993; Vandieken and Thamdrup, 2013; Hyun et al., 2017).

## Conclusion

Our approach complements earlier qualitative studies on microbial acetate assimilation in marine sediments, as we can now quantify the acetate uptake by distinct populations in high sensitivity and accuracy and determine their relative contribution to the total bacterial acetate uptake. By integrating whole-cell scintillography, phylogenetic identity and relative cell abundances we were able to show that not the common suspects such as the deltaproteobacterial SRB but rather diverse *Gammaproteobacteria* accounted for major acetate assimilation, particularly in oxic-suboxic sediments. Other populations abundant in marine sediments such as Acidobacteria, Bacteroidetes, and Planctomycetales have not been considered in our study but may also bear the potential to assimilate acetate in substantial amounts and thereby compete with SRB. Given the high *in situ* abundances of the identified populations in global sediments and the pivotal role of acetate as intermediate in organic matter degradation, *Gammaproteobacteria* and the Sva0081-MBG possibly play important roles in carbon mineralization in coastal sediments.

## Author Contributions

SD designed the research, performed the FISH and FACS, discussed the data, and conceived and edited the manuscript, and edited the manuscript. SL performed the incubations, microautoradiography, and measurements of bulk uptake. JS performed the VFA extractions and measurements. MM designed the research, performed the probe design, discussed the data, and conceived and edited the manuscript.

## Conflict of Interest Statement

The authors declare that the research was conducted in the absence of any commercial or financial relationships that could be construed as a potential conflict of interest.
