# Supplementary material for: Uncultured Gammaproteobacteria and Desulfobacteraceae Account for Major Acetate Assimilation in a Coastal Marine Sediment
Source: Front Microbiol. 2018 Dec 18;9:3124. doi: 10.3389/fmicb.2018.03124 (PMC6305295; doi:10.3389/fmicb.2018.03124)
Supplement: Supplementary file 1 [file Image_1.PDF]

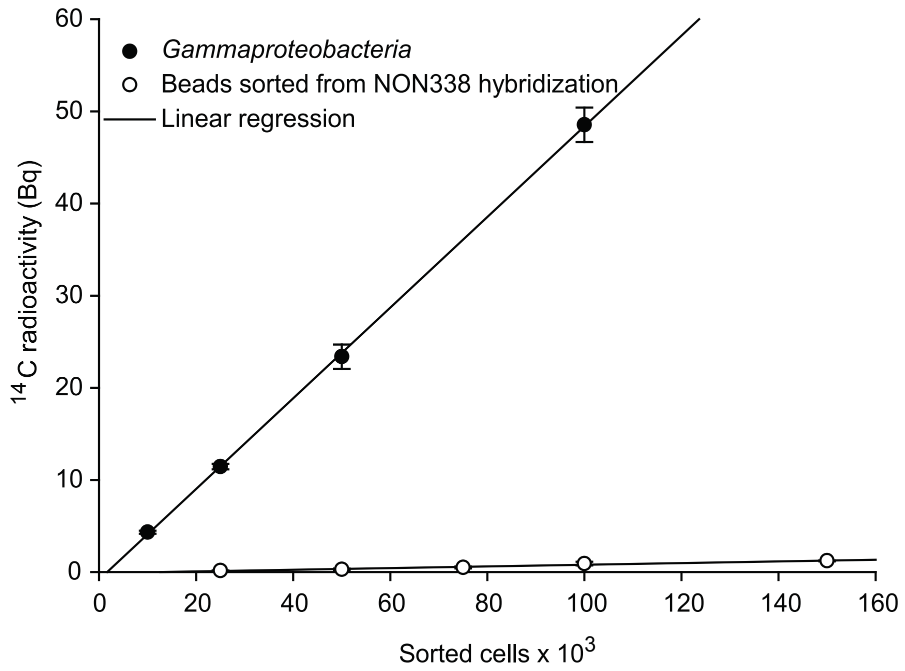

Fig. S1. Correlation of ^{14}C-carbon activity with abundances of flow-sorted cells of *Gammaproteobacteria* and fluorescent beads. To determine the unspecific background from ^{14}C-acetate incubations, sediments slurries were supplemented with fluorescent beads and hybridized with the negative control probe (NON338). Fluorescent beads were then flow-sorted before liquid scintillography. ^{14}C-carbon activity is given in Becquerel (Bq). Error bars indicate the standard deviation (SD) of triplicate flow-sorting.
