# Supplementary material for: Uncultured Gammaproteobacteria and Desulfobacteraceae Account for Major Acetate Assimilation in a Coastal Marine Sediment
Source: Front Microbiol. 2018 Dec 18;9:3124. doi: 10.3389/fmicb.2018.03124 (PMC6305295; doi:10.3389/fmicb.2018.03124)
Supplement: Supplementary file 2 [file Image_2.PDF]

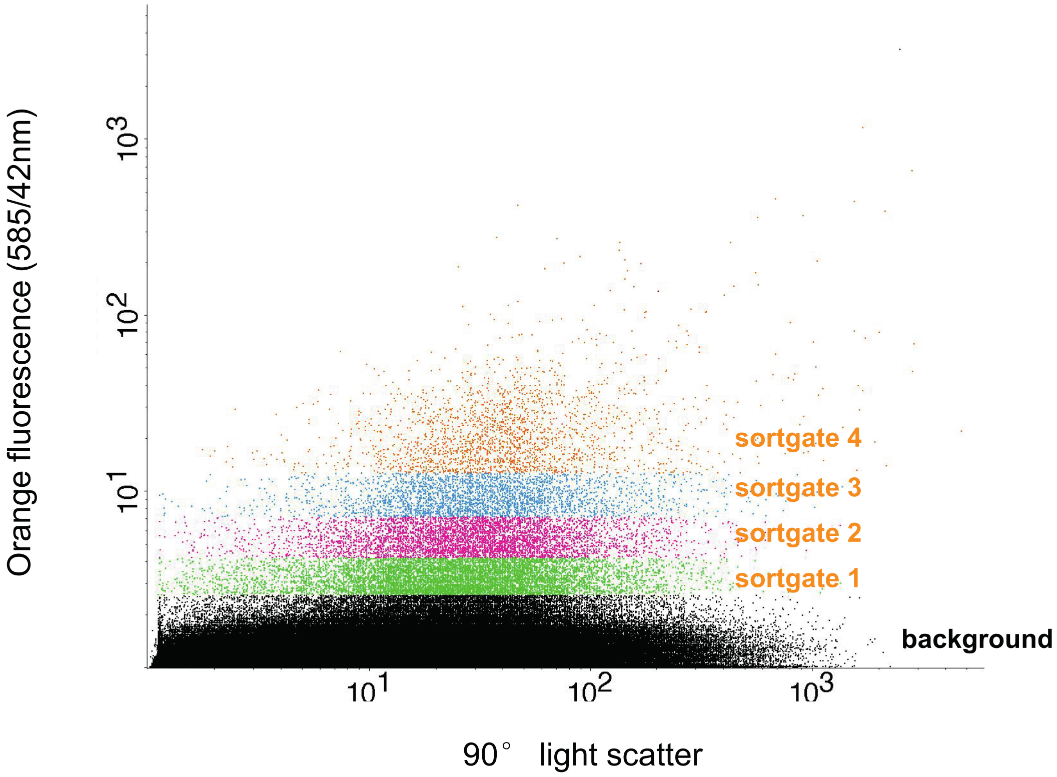

Fig. S2. Characteristic signatures of sediment samples stained with Nile Red and analysed by flow cytometry. Dot plot diagram of orange fluorescence plotted versus 90° light scatter. The gates used for cell sorting are indicated (sortgate 1-4).
