# Supplementary material for: Uncultured Gammaproteobacteria and Desulfobacteraceae Account for Major Acetate Assimilation in a Coastal Marine Sediment
Source: Front Microbiol. 2018 Dec 18;9:3124. doi: 10.3389/fmicb.2018.03124 (PMC6305295; doi:10.3389/fmicb.2018.03124)
Supplement: Supplementary file 3 [file Image_3.PDF]

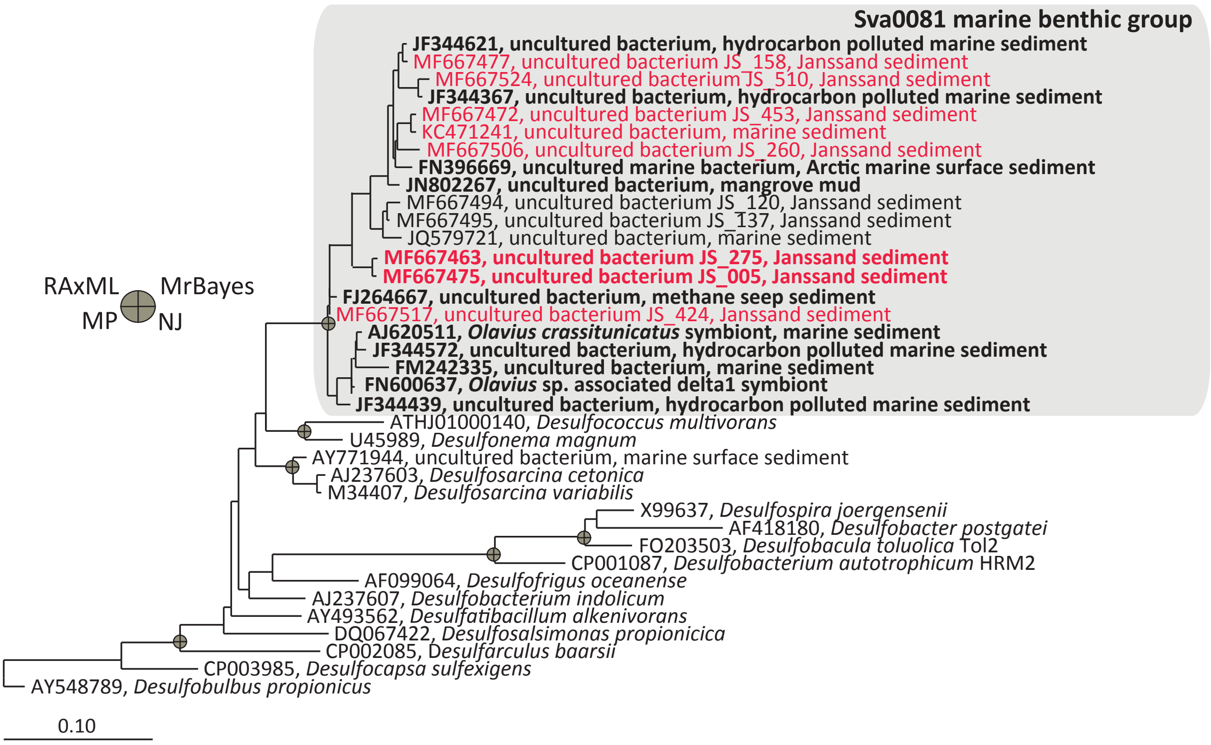

Fig. S3. Phylogenetic reconstruction of 16S rRNA gene sequences of selected *Desulfobacterales* based on RAxML. Only branching patterns supported by all 4 treeing methods are indicated. Partial Sva0081-MBG sequences retrieved from Janssand sediments are given in red. Sequences given in bold are targeted by the Sva0081-MBG specific probe DSS1431. Note that some partial sequences were too short to cover the target site of probe DSS1431. The scale bar refers to 10% sequence divergence.
