# Supplementary material for: Uncultured Gammaproteobacteria and Desulfobacteraceae Account for Major Acetate Assimilation in a Coastal Marine Sediment
Source: Front Microbiol. 2018 Dec 18;9:3124. doi: 10.3389/fmicb.2018.03124 (PMC6305295; doi:10.3389/fmicb.2018.03124)
Supplement: Supplementary file 4 [file Image_4.PDF]

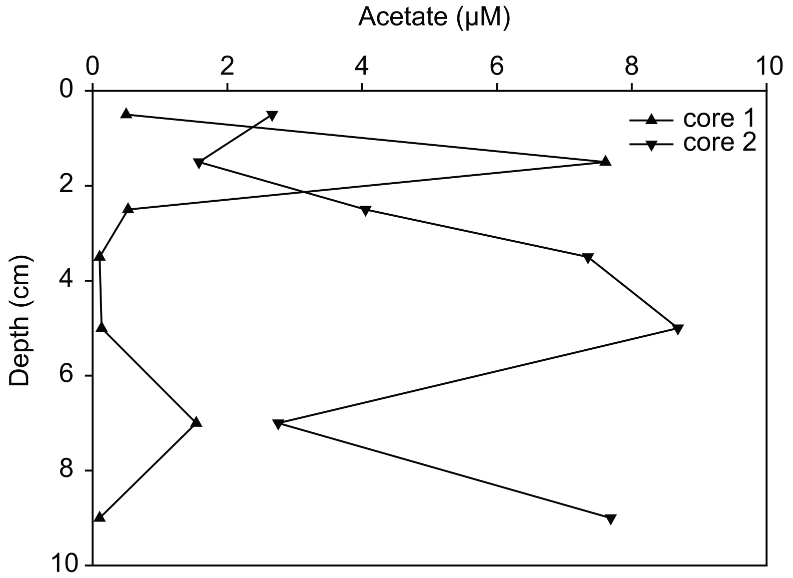

Fig. S4. *In situ* pore water concentrations of acetate in sediment cores sampled in duplicates at site Janssand in June 2009.
