# Supplementary material for: Uncultured Gammaproteobacteria and Desulfobacteraceae Account for Major Acetate Assimilation in a Coastal Marine Sediment
Source: Front Microbiol. 2018 Dec 18;9:3124. doi: 10.3389/fmicb.2018.03124 (PMC6305295; doi:10.3389/fmicb.2018.03124)
Supplement: Supplementary file 5 [file Image_5.PDF]

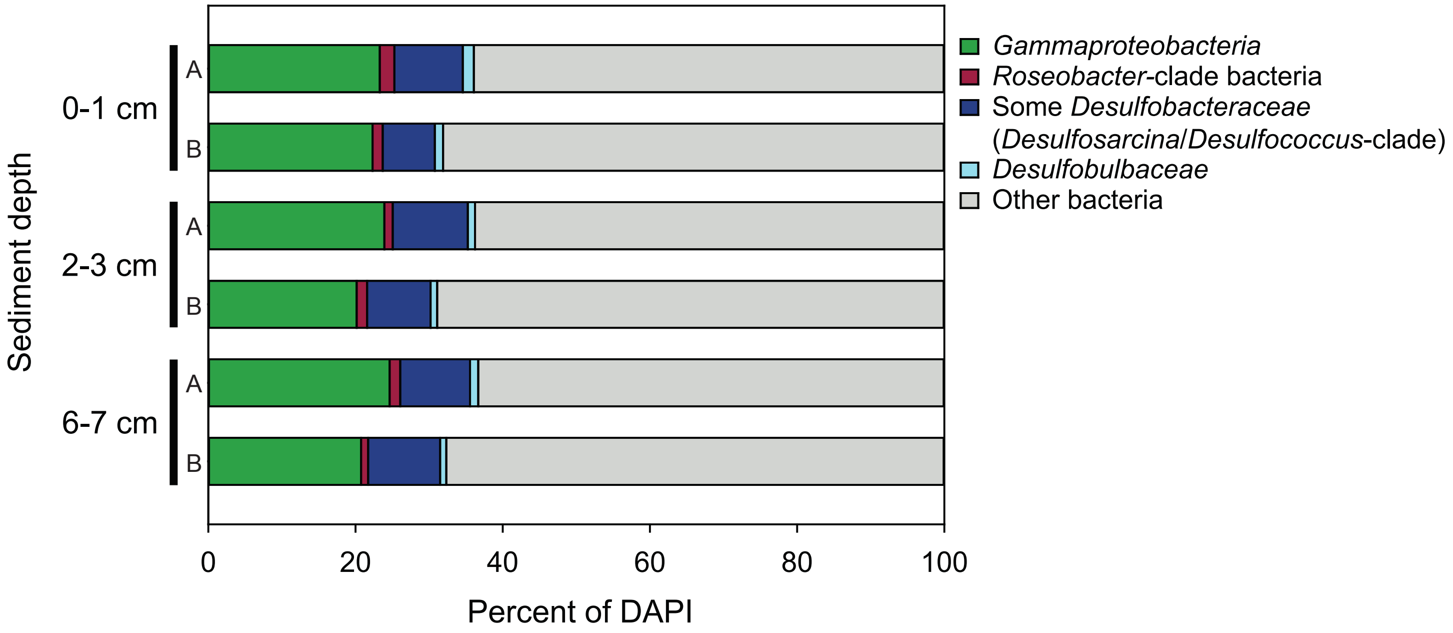

Fig. S5. Relative cell abundance over sediment depth in percent of total cell counts (DAPI) in two sediment cores (A, B) used for radiotracer incubations.
