# Supplementary material for: Uncultured Gammaproteobacteria and Desulfobacteraceae Account for Major Acetate Assimilation in a Coastal Marine Sediment
Source: Front Microbiol. 2018 Dec 18;9:3124. doi: 10.3389/fmicb.2018.03124 (PMC6305295; doi:10.3389/fmicb.2018.03124)
Supplement: Supplementary file 6 [file Table_1.PDF]

Supplementary Tables

Table S1. Horseradish peroxidase (HRP)-labelled oligonucleotide probes used for CARD-FISH. Helper and competitor oligonucleotides were not labelled.

Probe	Specificity	Sequence (5'-3')	FA (%)	Reference
EUB I-III	most Bacteria	GCWGCCWCCCGTAGGWGT	35	Amann *et al.* (1990) Daims *et al.* (1999)
NON338	Negative control	ACTCCTACGGGAGGCAGC		Wallner *et al.* (1993)
GAM42a*	most *Gammaproteobacteria*	GCCTTCCCACATCGTTT	35	Manz *et al.* (1992)
ROS537	*Roseobacter*-clade bacteria (RCB)	CAACGCTAACCCCCTCC	35	Eilers *et al.* (2000)
DSS658	many marine *Desulfobacteraceae* including the *Desulfosarcina/ Desulfococcus*-group	TCCACTTCCCTCTCCCAT	50	Manz *et al.* (1998)
DSB706	many marine *Desulfobulbaceae*	ACCGGTATTCCTCCCGAT	40	Loy *et al.* (2002)
Delta495 a-c*	most *Deltaproteobacteria*	AGTTAGCCGGTGCTTCCT<br>AGTTAGCCGGCGCTTCCT<br>AATTAGCCGGTGCTTCCT	35	Lücker *et al.* (2007)
DSS1431*	subgroup of Sva0081-MBG	GGTTTGCCCAACGACTTC	30	This study
cDSS1431a†	Competitor to probe DSS1431	GGTTTGCCCAACAACTTC	30	This study
cDSS1431b†	Competitor to probe DSS1431	GGTTAGCCCAACAACTTC	30	This study
cDSS1431c†	Competitor to probe DSS1431	GGTTCGCCCACCAACTTC	30	This study
cDSS1431d†	Competitor to probe DSS1431	AGTTTGCCCAACAACTTC	30	This study
cDSS1431e†	Competitor to probe DSS1431	GGTTGGCCCAACAACTTC	30	This study
DSS1431us‡	Helper to probe DSS1431	TGGTACAACCAACTCTCATGG	30	This study
DSS1431us‡	Helper to probe DSS1431	TTAGGCGCCTGCATCCCCGAA	30	This study
DSS1431us‡	Helper to probe DSS1431	TTAGGCGCCTGCATCCTGTAAA	30	This study

FA, formamide; * probes were used with published competitors; † competitor oligonucleotide; ‡ helper oligonucleotide

Table S2. Relative abundances of ^{14}C-acetate-assimilating cells as determined by microautoradiography and DAPI staining or hybridization with specific CARD-FISH probes in duplicate whole sediment cores incubated with ^{14}C-acetate.

**Sorted population**	**% of all MAR-positive cells** (core A/B)	**% MAR-positive of hybridized cells** (core A/B)
Gammaproteobacteria	*59/61*	19/19
Roseobacter-clade bacteria (RCB)	6/8	48/32
Deltaproteobacteria	8/4	9/7

Table S3. Average cell-specific ^{14}C-acetate assimilation rates of flow-sorted populations from duplicate whole core and slurry incubations. Cells were either labelled by CARD-FISH (16S rRNA) or by Nile Red-staining (polyhydroxyalkanoate, PHA).

	**Cell-specific assimilation rates (fg C cell^{-1} d^{-1})** core A/B						
**Sample type**	***Bacteria***	***Gamma-proteob.***	**RCB**	***Desulfobacteraceae***	Sva0081	***Desulfo-bulbaceae***	**PHA*** (min-max)
core incubations A/B:							
0-1 cm	3.8/1.3	5.5/3.9	9.7/7.8	3.3/2.5	n.d.	4.9/7.1	
2-3 cm	0.2/0.3	0.6/0.8	0.1/1.3	0.9/0.6	n.d.	1.3/3.0	
6-7 cm	0.2/0.2	0.3/0.2	0.4/0.5	0.3/0.2	n.d.	0.8/1.6	
slurry incubations A/B:							
oxic	3.5/5.8	11.9/17.6	3.8/5.3	1.1/1.2	2.7/2.8	2.5/3.0	1-26
anoxic	n.d.	n.d.	n.d.	n.d.	n.d.	n.d.	1-20

n.d, not determined; * polyhydroxyalkanoates stained with Nile red

References:

Amann, R.I., Binder, B.J., Olson, R.J., Chisholm, S.W., Devereux, R., and Stahl, D.A. (1990) Combination of 16S rRNA-targeted oligonucleotide probes with flow cytometry for analyzing mixed microbial populations. *Appl. Environ. Microbiol.* 56: 1919–1925.

Daims, H., Brühl, A., Amann, R., Schleifer, K.H., and Wagner, M. (1999) The domain-specific probe EUB338 is insufficient for the detection of all *Bacteria*: development and evaluation of a more comprehensive probe set. *Syst. Appl. Microbiol.* 22: 434–444.

Eilers, H., Pernthaler, J., Glöckner, F.O., and Amann, R. (2000) Culturability and *in situ* abundance of pelagic bacteria from the North Sea. *Appl. Environ. Microbiol.* 66: 3044–3051.

Loy, A., Lehner, A., Lee, N., Adamczyk, J., Meier, H., Ernst, J., *et al.* (2002) Oligonucleotide microarray for 16S rRNA gene-based detection of all recognized lineages of sulfate-reducing prokaryotes in the environment. *Appl. Environ. Microbiol.* 68: 5064–5081.

Lücker, S., Steger, D., Kjeldsen, K.U., MacGregor, B.J., Wagner, M., and Loy, A. (2007) Improved 16S rRNA-targeted probe set for analysis of sulfate-reducing bacteria by fluorescence *in situ* hybridization. *J. Microbiol. Methods* 69: 523–528.

Manz, W., Amann, R., Ludwig, W., Wagner, M., and Schleifer, K.-H. (1992) Phylogenetic oligodeoxynucleotide probes for the major subclasses of *Proteobacteria*: problems and solutions. *Syst. Appl. Microbiol.* 15: 593–600.

Manz, W., Eisenbrecher, M., Neu, T.R., and Szewzyk, U. (1998) Abundance and spatial organization of Gram-negative sulfate-reducing bacteria in activated sludge investigated by *in situ* probing with specific 16S rRNA targeted oligonucleotides. *FEMS Microbiol. Ecol.* 25: 43–61.

Wallner, G., Amann, R., and Beisker, W. (1993) Optimizing fluorescent *in situ* hybridization with rRNA-targeted oligonucleotide probes for flow cytometric identification of microorganisms. *Cytometry* 14: 136–143.
